# Early mortality in hip fracture patients admitted during first wave of the COVID-19 pandemic in Northern Italy: a multicentre study

**DOI:** 10.1186/s10195-021-00577-9

**Published:** 2021-04-05

**Authors:** Dante Dallari, Luigi Zagra, Pietro Cimatti, Nicola Guindani, Rocco D’Apolito, Federico Bove, Alessandro Casiraghi, Fabio Catani, Fabio D’Angelo, Massimo Franceschini, Alessandro Massè, Alberto Momoli, Mario Mosconi, Flavio Ravasi, Fabrizio Rivera, Giovanni Zatti, Claudio Carlo Castelli

**Affiliations:** 1 Reconstructive Orthopaedic Surgery and Innovative Techniques–Musculoskeletal Tissue Bank, IRCCS Istituto Ortopedico Rizzoli, Via G.C. Pupilli 1, 40136 Bologna, Italy; 2 IRCCS Istituto Ortopedico Galeazzi, Via R. Galeazzi 4, 20161 Milan, Italy; 3 Department of Orthopaedic Surgery, ASST Papa Giovanni XXIII, Piazza OMS 1, 24127 Bergamo, Italy; 4 Department of Orthopaedic Surgery, ASST Grande Ospedale Metropolitano Niguarda, Piazza Ospedale Maggiore 3, 20162 Milan, Italy; 5 Department of Orthopaedic Surgery, ASST Degli Spedali Civili di Brescia, Piazzale Spedali Civili 1, 25123 Brescia, Italy; 6 Department of Orthopaedic Surgery, Policlinico Universitario di Modena, Via del Pozzo 71, 41124 Modena, Italy; 7 Division of Orthopaedics and Traumatology, ASST Dei Sette Laghi, Department of Biotechnology and Life Sciences (DBSV), University of Insubria, Viale L. Borri 57, 21100 Varese, Italy; 8 ASST Gaetano Pini- CTO, Piazza A. Ferrari 1, 20122 Milan, Italy; 9 Department of Orthopaedic Surgery Ospedale Città Della Salute e Della Scienza, Università di Torino, Via G. Zuretti 29, 10126 Turin, Italy; 10 Department of Orthopaedic Surgery Ospedale San Bortolo, Viale F. Rodolfi 37, 36100 Vicenza, Italy; 11 Department of Orthopaedic Surgery, IRCCS Policlinico San Matteo di Pavia, Viale C. Golgi 19, 27100 Pavia, Italy; 12 Department of Orthopaedic Surgery, ASST Melegnano Martesana-Ospedale di Vizzolo Predabissi, Via Pandina 1, 20077 Vizzolo Predabissi, Italy; 13 Department of Orthopaedic Surgery Ospedale SS Annunziata, Via Ospedali 14, 12038 Savigliano, Italy; 14 Department of Orthopaedic Surgery ASST di Monza, Università Milano Bicocca, Via Pergolesi 33, 20900 Monza, Italy

**Keywords:** Proximal femur, Pertrochanteric fractures, Neck of the femur, Osteosynthesis, Hip arthroplasty, Cephalomedullary nail, SARS-CoV-2, Coronavirus, COVID-19

## Abstract

**Background:**

Treatment of hip fractures during the coronavirus disease 2019 (COVID-19) pandemic has posed unique challenges for the management of COVID-19-infected patients and the maintenance of standards of care. The primary endpoint of this study is to compare the mortality rate at 1 month after surgery in symptomatic COVID-positive patients with that of asymptomatic patients. A secondary endpoint of the study is to evaluate, in the two groups of patients, mortality at 1 month on the basis of type of fracture and type of surgical treatment.

**Materials and methods:**

For this retrospective multicentre study, we reviewed the medical records of patients hospitalised for proximal femur fracture at 14 hospitals in Northern Italy. Two groups were formed: COVID-19-positive patients (C+ group) presented symptoms, had a positive swab for severe acute respiratory syndrome coronavirus 2 (SARS-CoV-2) and received treatment for COVID-19; COVID-19-negative patients (C− group) were asymptomatic and tested negative for SARS-CoV-2. The two groups were compared for differences in time to surgery, survival rate and complications rate. The follow-up period was 1 month.

**Results:**

Of the 1390 patients admitted for acute care for any reason, 477 had a proximal femur fracture; 53 were C+ but only 12/53 were diagnosed as such at admission. The mean age was > 80 years, and the mean American Society of Anesthesiologists (ASA) score was 3 in both groups. There was no substantial difference in time to surgery (on average, 2.3 days for the C+ group and 2.8 for the C− group). As expected, a higher mortality rate was recorded for the C+ group but not associated with the type of hip fracture or treatment. No correlation was found between early treatment (< 48 h to surgery) and better outcome in the C+ group.

**Conclusions:**

Hip fracture in COVID-19-positive patients accounted for 11% of the total. On average, the time to surgery was > 48 h, which reflects the difficulty of maintaining normal workflow during a medical emergency such as the present pandemic and notwithstanding the suspension of non-urgent procedures. Hip fracture was associated with a higher 30-day mortality rate in COVID-19-positive patients than in COVID-19-negative patients. This fact should be considered when communicating with patients and/or their family. Our data suggest no substantial difference in hip fracture management between patients with or without COVID-19 infection. In this sample, the COVID-19-positive patients were generally asymptomatic at admission; therefore, routine screening is recommended.

**Level of evidence:**

Therapeutic study, level 4.

## Background

Hip fracture is one of the most common injuries among the elderly and is associated with a high mortality rate within 30 days after the injury event [1, 2]. The management of hip fracture is usually based on a diagnostic and therapeutic algorithm designed to reduce the risk of complications and to optimise resources. Various organisational models have been developed to identify modifiable risk factors [3] and perioperative risk. In hospitals throughout Northern Italy, perioperative assessment is standardised according to national guidelines. The goal is to prepare and treat patients within 48 h after presentation in the emergency department, except for patients judged ineligible for surgery (e.g. prolonged coagulation, organ failure, serious malnourishment). This objective is monitored by health agencies as a performance indicator of orthopaedic departments. Maintenance of a smooth workflow is both time-saving and of paramount importance for the delivery of care.

Prolonged time to surgery has been associated with higher mortality and short-term post-surgical complications [4, 5]. The coronavirus disease 2019 (COVID-19) pandemic has brought about major changes in the delivery of care: staff and resources have been redirected to essential services, drawing resources away from elective procedures and other departments [6]. During this pandemic period, there was a marked reduction in the number of paediatric emergencies and an increased proportion of proximal femoral fractures [7]. In compliance with national and World Health Organization (WHO) guidelines, elective procedures were suspended and non-urgent procedures were either cancelled or postponed [8]. This meant that a strategy to manage trauma patients safely had to be promptly devised [9–11].

During this period, patients were triaged in emergency departments on the basis of COVID-19 signs and symptoms along two different pathways: one for suspected COVID-19 infection and one for absence of infection. Salient presenting symptoms for COVID-19 were fever (body temperature ≥ 37.5°), cough, pharyngodynia, dyspnoea and dysgeusia [12, 13]. Patients with one or more symptoms underwent measurement of respiratory rate, arterial blood gas, peripheral oxygen saturation (SpO_2_) and chest X-ray. In cases of suspected COVID-19 infection, patients were admitted to a COVID-19 care unit and underwent a swab test for severe acute respiratory syndrome coronavirus 2 (SARS-CoV-2). An operating room specifically for COVID-19-positive patients was set up in nearly all hospitals [9, 10].

Here, we report the early mortality and morbidity in COVID-19-positive and COVID-19-negative patients treated for proximal femur fracture early during the first lockdown in Northern Italy due to the COVID-19 pandemic.

## Materials and methods

This retrospective multicentre study involving patients hospitalised for proximal femur fracture during lockdown (8 March through 4 May) was conducted in 14 hospitals located in four regions in Northern Italy (Lombardy, Veneto, Piedmont, Emilia-Romagna):

- Bergamo, Papa Giovanni XXIII Hospital; Brescia, Spedali Civili; Milan, Niguarda Hospital; Modena, University Hospital; Monza, San Gerardo University Hospital; Pavia, IRCCS Policlinico San Matteo Hospital; Turin, Città della Salute e della Scienza; Varese, Circolo e Fondazione Macchi Hospital; Vicenza, San Bortolo Hospital; Bologna, IRCCS Rizzoli Orthopaedic Institute; Milan, Gaetano Pini Orthopaedic Institute; Milan, IRCCS Galeazzi Orthopaedic Institute; Melegnano, Vizzolo Predabissi Hospital; Savigliano, SS Annunziata Hospital.

In all these hospitals, clearly defined pathways have been protocolled and made available to healthcare professionals who care for these patients. All staff have been specifically trained to don, doff and dispose of personal protective equipment (PPE) including masks, eye protection, double gloves, gowns, suits and caps. Surgical COVID-19-positive patients in transit through the theatre block were taken directly to a designated operating room which had to be marked with a clearly visible door sign. The patient’s transit to and from the COVID operating areas had to be as fast as possible. Therefore, in each hospital, the shortest possible predefined direct path was created away from other patients and people within the hospital to minimise the possibility of infection.

The study was approved by the ethics committee of the principal study centre (Bergamo, Number 31_21), which collected the data from the other centres in aggregated and anonymous form. All procedures were performed in accordance with the ethical standards of the institutional committee and the tenets of the 1964 Helsinki Declaration and its later amendments.

The medical records were reviewed, and the following data were extracted: patient age and sex; result of the nasopharyngeal swab and real-time polymerase chain reaction (rPCR)-test for SARS-CoV-2 on hospital admission, fracture type and location according to the Arbeitsgemeinschaft für Osteosynthesefragen Foundation/Orthopaedic Trauma Association (AO/OTA) classification system, comorbidities based on the Charlson Comorbidity Index and the Clinical Frailty Scale, the American Society of Anesthesiologists (ASA) score, the type of surgery (internal fixation and open reduction, hemiarthroplasty or total hip arthroplasty), length of hospital stay (LOS) and perioperative complications.

Inclusion criteria were a proximal femur fracture, age ≥ 18 years and treatment received between 8 March and 4 May 2020. Based on the AO/OTA classification for adults, proximal femur fractures (AO-31) were grouped into medial (AO-31B + C) and lateral (AO-31A), including pertrochanteric (AO-31A.1 and AO-31A.2) and subtrochanteric (AO-31A.3) fractures. Exclusion criteria were hip fracture treated non-operatively, age < 18 years, periprosthetic fractures, refusal to complete a telephone follow-up interview or lost at 1 month follow-up. At 1 month of follow-up, patients were contacted by phone/telemedicine or underwent clinical examination.

For the present study, a patient was categorised as COVID-19^1^ positive (C+) if, during hospital stay, all of the following criteria were met: positive SARS-CoV-2 swab test; symptomatic^2^; medical therapy for COVID-19 infection. Otherwise, a patient was categorised as COVID-19 negative (C−). The outcomes in terms of early mortality and morbidity between the two groups (C+ versus C−) were compared.

### Statistical analysis

Sample distributions of continuous data were tested for normality with the Kolmogorov–Smirnov and Anderson–Darling tests. Accordingly, continuous normal variables were described as mean ± standard deviation (m ± s) and compared by using the unpaired Student’s *t*-test. Dichotomous categorical variables were expressed as frequencies and percentage and compared by Pearson’s *χ*^2^ or Fisher’s exact test, according to minimum frequency. Rank tests were used for non-normally distributed variables and for ordinal ones. All tests were two-tailed. Data were considered statistical significant at *p* < 0.05. Statistical analyses were performed with GraphPad Prism version 6.0 software (www.graphpad.com) and Microsoft Excel, Microsoft Corporation, USA.

## Results

Of the 1390 patients treated in a trauma care department for any reason, 477 (35% of all hospital admissions) presented with a proximal femur fracture and were included in the study; 53/477 (11%) were defined as C+ based on the inclusion criteria; and 12/53 (23%) presented signs and symptoms of COVID-19 infection at admission, while the others developed symptoms after admission. Table 1 presents patient demographics; the mean age was > 80 years, and the majority were in ASA class 3 (51% in the C+ group and 50% in the C− group). The only significant difference between the two groups was patient sex. There was a similar distribution of fracture types (pertrochanteric, medial neck, subtrochanteric) in both groups (Table 2). The average time between admission and surgery was 2.3 ± 0.4 days in the C+ group and 2.8 ± 0.1 days in the C− group (Table 3).

**Table 1:** Demographic characteristics

Demographics	C+ (*n* = 53) m ± s (CI_95_)	C– (*n* = 424) m ± s (CI_95_)	*p*	Difference of the means (CI 95)
Age (mean ± SD)	83.3 ± 0.9 (83.05 to 83.55)	81.2 ± 0.5 (81.15 to 81.25)	0.156^a^	2.1 (2.26 to 1.94)
Sex, *n* (%)			0.044 ^a^	
Male	5/53 (9%)	91 (21.5%)		
Female	48/53 (91%)	333 (78.5%)		
CCI (mean ± SD)	6.0 ± 0.3 (5.92 to 6.08)	5.5 ± 0.1 (5.49 to 5.51)	0.093 ^a^	0.5 (0.46 to 0.54)
CCI ≥ 4, *n* (%)	47/53 (88.7%)	359 (84.6%)		
CFS (mean ± SD)	5.3 ± 0.2 (5.24 to 5.36)	5.2 ± 0.1 (5.19 to 5.21)	0.612 ^a^	0.1 (0.07 to 0.13)
CFS ≥ 4, *n* (%)	42/53 (79%)	318 (75.0%)		
ASA	2.7 ± 0.1 (2.67 to 2.73)	2.6 ± 0.1 (2.59 to 2.61)	0.427 ^a^	
Median (mode, IQR_25–75_)	3 (3; 2–4)	3 (3; 2–4)	0.2 ^b^	
ASA = 1, *n* (%)	1/52 (2%)	9/370 (2%)		
ASA = 2, *n* (%)	19/52 (37%)	152/370 (41%)		
ASA = 3, *n* (%)	27/52 (51%)	184/370 (50%)		
ASA = 4, *n* (%)	5/52 (10%)	25/370 (7%)		
ASA = 5, *n* (%)	0/52 (0%)	0 /370 (0%)		
ASA = 6, *n* (%)	0/52 (0%)	0/370 (0%)		
ASA = n.a. (%)	1/53 (2%)	54/424 (13%)		

m: mean of the sample; s: standard deviation of the sample; CI_95_: confidence interval (95%). IQR_25–75_: inter-quartile range, from 25 to 75%; n.a.: not available data

C+: patients with COVID-19; C−: control group; see definitions in *Materials and methods*

^a^heteroscedastic two-tailed *t*-test

^b^Mann–Whitney test

**Table 2:** Type of fracture

Injury information	C+ (*n* = 53) Nr (%); CI_95_	C− (*n* = 424) Nr (%)–CI_95_	*p; Δm (CI*_*95*_*)*
Type of proximal femur fracture, *n* (%)			
Pertrochanteric fracture (AO-31A.1/2)	22/53 (42%); 0.29–0.55	221/424 (52%); 0.47–0.57	1.53 CI 0.86–2.74
Medial fracture (AO-31B + C)	29/53 (55%); 0.4–0.67	191/424 (45%); 0.40–0.50	0.68 CI 0.38–1.20
Subtrochanteric fracture (AO-31A.3)	2/53 (4%); 0.01–0.13	12/424 (3%); 0.02–0.05	0.74 CI 0.16–3.41

C+: patients with COVID-19; C−: control group; see definitions in *Materials and methods*. Δm = diffence of the means (m_C+_ -m_C−_). AO: Arbeitsgemeinschaft für Osteosynthesefragen

**Table 3:** Time from admission to surgery

Surgical information	C+ (*n* = 53) m ± s; CI_95_	C− (*n* = 424) m ± s; CI_95_	*p*; *Δm (CI*_*95*_*)*
Time from admission to surgery [days]	2.3 ± 0.4; 2.19–2.41	2.8 ± 0.1; 2.79–2.81	0.693; −0.5 (−0.55 to −0.45)

m: mean of the sample; s: standard deviation of the sample; CI_95_: confidence interval (95%), Δm: difference of the means (m_C+_ − m_C−_). C+: patients with COVID-19; C−: control group; see definitions in *Materials and methods*

Postoperative morbidity and mortality were higher in the C+ group and concerned post-operative complications and mortality rate during the stay in hospital and the first post-operative month (Table 4). As expected, the 1-month mortality rate was higher among the C−patients operated later, than in those operated within 48 h of admission. No such association was found for the C+ patients. The 1-month mortality rate for those operated within 48 h of admission was 23.8%, and 9.1% for those operated after 48 h. However, the small sample size of the subgroup of C+ operated after 48 h reduced statistical power and does not allow statistical inferences to be drawn regarding this population (Table 4).

**Table 4:** Morbidity and mortality compared between C+ and C−

Hospital quality measures	C+ (*n* = 53); CI_95_	C− (*n* = 424); CI_95_	*p*; OR *(CI*_*95*_*)*
Post-operative complications (tot)	34 (64%); 0.51–0.76	205 (48%); 0.44–0.53	***0.026***; 0.52 (0.29–0.95)
Acute anaemia	16 (30%)	138 (33%)	
Pneumonia	6 (11%)	7 (2%)	
Other respiratory complications	6 (11%)	8 (2%)	
Acute heart failure	3 (6%)	9 (2%)	
Urinary tract infection	2 (4%)	7 (2%)	
Acute kidney failure	1 (2%)	2 (1%)	
Sepsis	0	3 (1%)	
Pulmonary thromboembolism	0	2 (1%)	
Ictus cerebri	0	2 (1%)	
Other minor complications	0	27 (7%)	
Mortality, *n* (%)			
Inpatient post-operative mortality	8/53 (15%); 0.08–0.27	7/424 (2%); 0.01–0.03	*** < 0.0001***; 0.09 (0.03–0.27)
Outpatient mortality (30-day)	12/53 (23%); 0.13–0.36	20/424 (5%);0.03–0.07	***0.008***; 0.17 (0.08–0.37)
Mortality (*t* < 48 h)	10/42 (24%); 0.13–0.39	15/306 (5%); 0.03–0.08	*** < 0.0001***; 0.16 (0.07–0.40)
IF	7/10	9/15	
HA	3/10	6/15	
THA	0	0	
Mortality (*t* ≥ 48 h)	1/11 (9%); 0.02–0.38	5/118 (4%); 0.02–0.10	0.420; 0.44 (0.05–4.17)
IF	0	4/5	
HA	1/11	1/5	
THA	0	0	

Statistically significant differences (*p* < 0.05) are expressed as bold italic data

*t* < or ≥ 48 h: treatment time from admission. OR: odds ratio. C+: patients with COVID-19; C−: control group; see definitions in *Materials and methods*

There was no association between type of surgery (reduction and fixation compared with arthroplasty) and 30-day mortality rate, which was considerably higher in the C+ group (Table 5). The mean length of hospital stay (LOS) was 10.9 ± 0.3 days for the C− group and 14.7 ± 1.5 days for the C+ group, respectively; the difference between the two groups was 4 days. The LOS was longer for the C+ group after all types of surgery, and the difference was statistically significant only for internal fixation.

**Table 5:** Internal fixation versus prosthetic implant for C+ versus C−

Variable	IF			Prostheses (HA + THA)		
	C+ (*n* = 24)	C− (*n* = 243)	*p* OR *(CI*_*95*_*)*	C+ (*n* = 22_HA_ + 7_THA_)	C– (*n* = 127_HA_ + 54_THA_)	*p* OR *(CI*_*95*_*)*
Tot number of patients (%)	24/53 (45%)	243/424 (57%)	0.107	29/53 (55%)	181/424 (43%)	0.107
CI_95_	0.33–0.59	0.53–0.62	1.62 (0.91–2.88)	0.41–0.67	0.38–0.47	0.62 (0.35–1.09)
Age (mean ± SD)	84.3 ± 1.3	81.7 ± 0.7	0.274	82.5 ± 1.4	80.5 ± 0.7	0.282
CI_95_	83.75–84.85	81.61–81.79	2.6 (2.28–2.92)	81.97–83.03	80.40–80.60	2.00 (1.67–2.33)
ASA (mean ± SD)	2.7 ± 0.1	2.6 ± 0.1	0.301	2.6 ± 0	2.6 ± 0.1	0.951
Median (IQR_25–75_)	3 (2–4)	3 (2–4)	0.431^a^	3 (2–4)	3 (2–4)	0.752^a^
30-day mortality	8/24 (33%)	13/243 (5%)	***0.0002***	4/29 (14%)	7/181 (4%)	***0.048***
CI_95_	0.18–0.53	0.03–0.09	0.11 (0.04–0.31)	0.05–0.31	0.02–0.08	0.25 (0.07–0.92)

Statistically significant differences (*p* < 0.05) are expressed as bold italic data

m: mean of the sample; s: standard deviation of the sample; CI_95_: confidence interval (95%); Δm: difference of the means (m_C+_ – m_C−_)

^a^Mann–Whitney. C+: patients with COVID-19; C−: control group; see definitions in *Materials and methods*

Most patients from both groups were discharged to intensive rehabilitation services or dedicated rehabilitation facilities; 5% of the C+ group patients were discharged home, compared with 41.2% of the C− group patients (Table 6). All patients received oxygen therapy (1/53 invasive ventilation, 4/53 non-invasive ventilation, 48/53 oxygen support without ventilation); 23/53 received antiviral drugs, 15/53 corticosteroids, 40/53 hydroxychloroquine; none received monoclonal antibodies^3^.

**Table 6:** Hospital quality measures

Hospital quality measures [days]	C+ (m ± s; CI_95_)	C− (m ± s; CI_95_)	*p*	*Δm (CI*_*95*_*)*
Length of stay (LOS)	14.7 ± 1.5; 14.29 to 15.11	10.9 ± 0.3; 10.87 to 10.93	**0.001**	3.8 (3.64 to 3.96)
LOS associated with type of surgery				
IF (C+ 24/53; C− 243/424)	13.8 ± 2.6; 12.70–14.90	9.8 ± 0.3; 9.76–9.84	**0.002**	4.0 (3.66–4.34)
HA (C+ 22/53; C− 127/424)	15.6 ± 1.9; 15.08–16.12	12.4 ± 0.8; 12.32–12.48	0.150	3.2 (3.00–3.40)
THA (C+ 7/53; C− 54/424)	14.0 ± 4.0; 12.90–15.10	11.7 ± 1.3; 11.58–11.82	0.649	2.3 (1.78–2.82)
Discharge type, *n* (%)				
IHR and RSA	38/40 (95%) 0.83–0.99	238/413 (58%) 0.53–0.62	**0.002**	0.07 (0.02–0.30)
Home	2/40 (5%) 0.01–0.17	175/413 (42.3%) 0.38–0.47	** < 0.0001**	13.97 CI 3.33–58.69
Missing data	13/53	11/424		

m: mean of the sample; s: standard deviation of the sample; CI_95_: confidence interval (95%); Δm: difference of the means (m_C+_ – m_C−_); C+: patients with COVID-19; C−: control group; see definitions in *Materials and methods*

## Discussion

Here, we report the short-term outcomes of patients with COVID-19 infection treated for a proximal femur fracture early during the COVID-19 pandemic in Northern Italy. Few patients were operated within 48 h of hospital admission, as envisaged by the standardised organisational model [14]. This delay could have been due to a variety of reasons, such as patients’ medical needs (C+ patients required more comprehensive pre-operative assessment) and organisational problems (time needed to equip an operating room for COVID-19 patients, reduced staff and operating room time slots, delays in hospital workflow). Nevertheless, with the limitations of the small C+ sample and the short follow-up, it would seem that the delay in surgical treatment is not accompanied by a lower mortality rate in C+ patients. The high risk associated with severe respiratory dysfunction and pneumonia secondary to COVID-19 infection may be a contraindication to urgent hip fracture surgery in COVID-19-positive patients, and pulmonary complications are known to contribute to post-operative morbidity [15–17].

No conclusions about indications to treat or not to treat a proximal femur fracture in patients with concurrent COVID-19 infection can be drawn from the present data. Furthermore, the severity of disease differed in the C+ group patients. Numerous studies have reported a high short-term mortality rate in COVID-19-positive patients with a proximal femur fracture [18–20]. To date, there is no gold standard for surgical indications and timing of surgery for such patients.

Egol et al. reported a 30-day mortality rate in their cohort of 138 patients: 53% COVID-19-positive and 5.6% COVID-19-negative [18]. Maniscalco et al. [19] reported outcomes in 32 COVID-19-positive hip fracture patients treated surgically: 43.8% (14/32) mortality rate within 21 days. Our data from a larger patient population share this observation. Despite the similar comorbidity profiles, the 30-day mortality rate was higher for the C+ group than the C− group. In their study, Kayani et al. [20] underlined the risk factors of smoking status and multiple (> 3) comorbidities.

Moreover, patients with a proximal femoral fracture are often frail because of co-occurring complications [21, 22]. The higher 1-month mortality rate observed for the C+ group may be explained by the severity of the COVID-19-related complications that these patients developed during the peri-operative period. The prevention of major post-operative complications in elderly COVID-19-positive patients with proximal femur fracture calls for a multidisciplinary approach (orthopaedics, anaesthesiology, geriatrics) to manage these high-risk patients.

We observed that the longer LOS recorded for the C+ group patients in hospital and dedicated rehabilitation facilities did not reduce the 30-day mortality rate. Poor outcomes after surgical treatment have been reported. The C+ group had a greater need for intensive care, also while undergoing surgery during the asymptomatic incubation period [23]. Efforts should be undertaken to improve the medical and surgical treatment of elderly COVID-19-positive patients with a proximal femur fracture. We noted a higher prevalence of women in the C+ group. Previous studies found no association between infection rate and sex, although the prognosis seems to be different [1, 11, 24].

The limitations of the present study include its retrospective design and the small number of C+ group patients. A small sample size results in reduced statistical power and may result in a lack of statistical representation of a phenomenon and its real distribution in the population.

A low statistical power can lead to overestimates of effect size and low reproducibility of results.

This multicentre study was created to collect as much data as possible and to observe in an evidence-based manner the mortality and complication rates in C+ and C− patients operated on for proximal femur fracture during the lockdown in the first COVID-19 wave. To the best of our knowledge, this is the largest cohort of C+ patients described in this field of orthopaedics and traumatology during the pandemic.

The inclusion criteria for the C+ group were a positive SARS-CoV-2 swab, symptoms and therapy, which may have led us to underestimate the number of COVID-19-positive patients. Asymptomatic but potentially COVID-19-positive patients were included in the C− group since asymptomaticity has not been considered relevant from the point of view of mortality, along with those with false negative swab tests; so, again, the differences between the C+ and the C− group may have been underestimated. These limitations notwithstanding, our data provide a picture of the real-world situation early during the pandemic when screening strategies had not yet been implemented. To the best of our knowledge, this is the largest cohort of patients described during the first wave of the pandemic. Also, the study’s multicentre design ensured the collection of data from different types of centres throughout the northern part of the country hardest hit by the pandemic in the spring of 2020.

In conclusion, COVID-19 infection was associated with higher 1-month mortality in patients with a proximal femur fracture. Despite the small size of the subgroup of C+ patients operated within 48 h and despite its numerical heterogeneity compared with the corresponding subgroup of C−patients operated within 48 h, it would seem that surgery within 48 h does not associate with a lower mortality in C+ patients. Future studies will be necessary to corroborate this data. The co-occurrence of COVID-19 infection and proximal femur fracture was infrequent early during the pandemic. Further data are needed to evaluate surgical indications and optimal timing, together with type and timing of medical intervention to reduce mortality rates.

## Data Availability

The datasets used and analysed during the current study are available from the database manager (NG) on reasonable request.

## Abbreviations

COVID-19: Coronavirus disease 2019 (COVID-19)
C+: COVID-19-positive
C−: COVID-19-negative
SARS-CoV-2: Severe acute respiratory syndrome coronavirus 2
WHO: World Health Organization
SpO2: Peripheral oxygen saturation
ASA: American Society of Anesthesiologists
LOS: Length of hospital stay

## Footnotes

1 According to the WHO, the virus responsible for COVID-19 is SARS-CoV-2 [8].

2 Clinical manifestations of COVID-19 range from mild pharyngorhinitis and anosmia/ageusia to severe interstitial pneumonitis and respiratory impairment; most had acute respiratory insufficiency needing medical attention including oxygen support [12].

3 Due to data dispersion in the subgroups, heterogeneity of medical therapy, and sample size, further statistical analysis to stratify the risk according to the medical therapy was not undertaken.
